# Gene therapy for p16-overexpressing cells

**DOI:** 10.18632/aging.101422

**Published:** 2018-04-19

**Authors:** Marco Demaria

**Affiliations:** 1 University of Groningen, European Institute for the Biology of Aging (ERIBA), University Medical Center Groningen (UMCG), Groningen, The Netherlands

**Keywords:** aging, senolytics, cellular senescence, cancer, p16

p16^Ink4a^ (p16) is an important tumor suppressor which is upregulated in senescent cells and in aged tissues. p16 acts as an inhibitor of the interaction between Cyclin-Dependent Kinases (CDK) 4/6 and CyclinD1 leading to the activation of retinoblastoma protein (RB). Consequently, active RB interferes with the translocation of E2F1 into the nucleus and arrests cells in the G1-S phase of the cell cycle [1].

In cancer cells with mutations in RB or CDK4/6, p16 is normally overexpressed but unable to induce cell cycle arrest. p16 -overexpressing cancer cells are found in different types of carcinomas and are considered highly aggressive and invasive [2].

Several drugs in recent years have been shown to have ‘senolytic’ properties (i.e. being toxic for senescent cells) and to remove p16^+^ cells from a variety of tissues [3]. Among these compounds, ABT-737 and its orally-available analogue ABT-263 target the anti-apoptotic proteins BCL-2, BCL-W and BCL-XL, considered essential pro-survival players in senescent cells. The effects of these compounds in mice almost completely overlap with a suicide gene strategy activated by the p16 promoter, thus suggesting specific targeting p16^+^ cells [3]. However, when we tested ABT-737 and ABT-263 against p16-overxpressing murine sarcomas we failed to observe any toxicity, despite p16^+^-cancer cells upregulating both BCL-2 and BCL-XL [4]. These data could be interpreted in 3 ways: 1) ABT compounds are specifically active against non-proliferating p16+-cells; 2) the efficacy of ABTs requires upregulation of BCL-W, which we have recently shown being a common feature of senescent cells [5]; 3) ABTs act independently of p16 expression levels. The latter hypothesis would represent a critical issue, as p16 is used as a major readout for the efficacy of senotherapies.

Since we have recently developed an inducible suicide gene regulated by the full p16 promoter [6], we have then studied whether the use of this strategy could be effective against p16^+^ tumors. Indeed, most p16 -overexpressing cancer cells were efficiently eliminated by the activation of the suicide gene in both culture and *in vivo* conditions [4]. These data suggest that p16 upregulation is maintained by active transcription, possibly mediated by emergency signaling pathways attempting to restrain cellular proliferation.

Our study supports the idea that the overexpression of oncosuppressors could be exploited for interventions against cancer. While we studied a specific context in which p16 is present in its wild-type form, this strategy could potentially work in situations of overexpression (by transcriptional regulation) of mutated forms, which is a common feature of cancer cells. On this line, similar strategies against additional oncosuppressors such as p14 and p53 could be effective.

In parallel, it will be of interest to understand whether a p16 -based suicide gene therapy could be used in other contexts. Studies in transgenic mouse models have shown that elimination of p16^+^ cells using suicide genes can significantly delay the onset and progression of a number of age-related pathologies, eventually leading to lifespan extension [7]. Whether a similar strategy could be used for human interventions is still matter of debate. Despite significant efforts and resources have been spent in the last two decades for the development of suicide gene therapies, particularly for cancer treatment, none of these therapies have been approved by regulatory agencies. Indeed, there are still a number of major limitations preventing the feasibility of suicide gene strategies for human treatment. First, damaged or mutated cells responsible to promote onset and progression of disease might be heterogeneous and might not express the same markers, thus limiting the efficacy of a therapy based on interfering with specific genes. Second, current suicidal strategies might not be entirely safe. For example, one of the most used suicide gene is the herpex simplex virus thymidine kinase (HSV-tk), shown to have significant bystander effects. Third, the delivery methods for genes in vivo are highly inefficient and not yet entirely safe to use in humans.

The advent of technical and intellectual breakthroughs overcoming these limitations could potentially resolve in an innovative, accessible and adaptable technology for the cure of many diseases.

## References

[1] Hernandez-Segura A, et al. Trends Cell Biol. 2018. 10.1016/j.tcb.2018.02.001 29477613

[2] Romagosa C, et al. Oncogene. 2011; 30:2087–97. 10.1038/onc.2010.614 21297668

[3] Childs BG, et al. Nat Rev Drug Discov. 2017; 16:718–35. 10.1038/nrd.2017.116 28729727 PMC5942225

[4] Kohli J, et al. Oncotarget. 2017; 9:7274–81. 10.18632/oncotarget.23752 29484109 PMC5800901

[5] Hernandez-Segura A, et al. Curr Biol. 2017; 27:2652–2660.e4. 10.1016/j.cub.2017.07.033 28844647 PMC5788810

[6] Demaria M, et al. Dev Cell. 2014; 31:722–33. 10.1016/j.devcel.2014.11.012 25499914 PMC4349629

[7] Wang D, Gao G. Discov Med. 2014; 18:151–61. 25227756 PMC4440458

